# Undecylprodigiosin Induced Apoptosis in P388 Cancer Cells Is Associated with Its Binding to Ribosome

**DOI:** 10.1371/journal.pone.0065381

**Published:** 2013-06-14

**Authors:** Ping Liu, Yuan-yuan Wang, Xin Qi, QianQun Gu, Meiyu Geng, Jing Li

**Affiliations:** 1 Key Laboratory of Marine Drugs, Ministry of Education, School of Medicine and Pharmacy, Ocean University of China, Qingdao, China; 2 Analysis and Testing Center, Yantai Institute of Coastal Zone Research, Chinese Academy of Sciences, Yantai, China; 3 Key Laboratory of Drug Research, Shanghai Institute of Materia Medica, Chinese Academy of Sciences, Shanghai, China; INRS, Canada

## Abstract

Prodigiosins (PGs) are a family of natural red pigments with anticancer activity, and one member of the family has entered clinical phase II trials. However, the anticancer mechanisms of PGs remain largely unclear. This study was designed to investigate the molecular basis of anticancer activity of UP, a derivative of PGs, in P388 cells. By introducing pharmacological inhibitors and utilizing a variety of analytical approaches including western blotting, flow cytometry and confocal laser microscopy, we found that UP inhibited proliferation of P388 via arresting cells at G2/M phase and inducing cells apoptosis, which was related to the activation of P38, JNK rather than ERK1/2 signaling. ROS regeneration and acidification in cells appear not involved in UP induced apoptosis. Furthermore, utilizing mass spectrometry, sucrose density gradient fractionation and immunofluorescence staining, we discovered that UP was apparently located at ribosome. These results together indicate that ribosome may be the potential target of UP in cancer cells, which opened a new avenue in delineating the anticancer mechanism of PGs.

## Introduction

**Figure 1:**
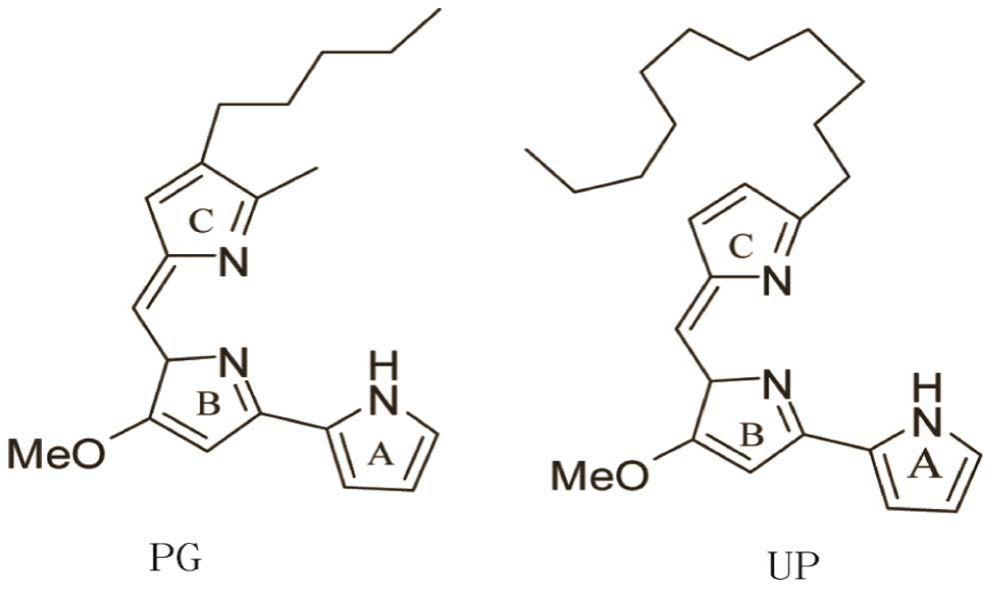
Structures of prodigiosin (PG) and Undecylprodigiosin (UP).

Prodigiosins (PGs) are a family of natural red pigments, structurally characterized by a common pyrrolylpyrromethene skeleton with varying side chains. PGs, originally isolated from Serratia by Amak in 1929, are composed of prodigiosin (PG), prodigiosin 25-C (PG 25-C), metacycloprodigiosin (MP), cycloprodigiosin (CPrG) and undecylprodigiosin (UP), etc. PGs are the secondary metabolites of various bacteria with various biological activities such as anti-microbial, anti-malarial, immunosuppressive and anticancer. The structures of PG and UP are shown in Figure 1 [1], [2].

Increasing studies have suggested the anticancer activity of PGs. It has been reported that PGs induces apoptosis in haematopoietic, gastrointestinal, breast and lung cancer cells whereas non-toxic to non-malignant cells [3]–[5]. Currently, a PGs derivative GX15-070 has entered clinical phase II trials for its anticancer activity [6].

Growing studies have been conducted to reveal the molecular targets of PGs to gain insights into its anticancer efficacy, but the findings revealed great discrepancies in different cellular context or using individual compounds. PGs have been reported to trigger signaling pathways possibly through induction of DNA double-strand breaks and/or neutralization of pH gradients, which leads to cell cycle alternations and apoptosis. Janus tyrosine kinase 3 (Jak3) that associates with IL-2R upon activation was also suggested to be the molecular target for PGs in gastric cancer cells [7]. Recently, M. Espona-Fiedler identified the mammalian target of rapamycin (mTOR) as a candidate molecular target of PGs in melanoma cells [8]. Nevertheless, the molecular mechanism of PGs remains largely unclear.

We have extracted UP from the fermentation broth of a sponge Mycale plumose-derived actinomycete *Saccharopolyspora sp.Nov*, and found that this compound exhibited significant cytotoxic activity against five cancer cell lines, among which the impact on murine leukemia P388 was the most evident [9]. In the present study, we attempted to reveal the molecular basis of UP on its anticancer activity in P388 cells. The results indicate that UP inhibits proliferation of P388 by inducing G2/M phase arrest and apoptosis, which was related to the activation of P38, JNK rather than ERK1/2 signaling. The ribosome appears the potential target of UP. This study provides molecular basis to further elucidate the anticancer mechanism of PGs in the future.

## Materials and Methods

### Reagents

UP and PG were provided by Professor Gu (School of Medicine and Pharmacy, Ocean University of China, Qingdao, China). Their purities were >99.5%. 4,6-diamidino-2-phenyllindile (DAPI), propidium iodide (PI). Fetal bovine serum (FBS) was purchased from GIBCO (Gaithersburg, MD). An enhanced chemiluminescence (ECL) kit was purchased from PIERCE(Holmdel, NJ).U0126, SP60012, SB203580, SRB, DCFH-DA, BCECF AM, AO were all purchased from Beyotime Institute of Biotechnology (Jiangsu, China). PARP, Cyt C, Caspase-3, Caspse-8, Caspase-9, β-actin, p-AKT, AKT, p-ERK, ERK, p-JNK, JNK, p-P38 and P38 antibody were purchased from Cell Signaling (Beverly, MA). Ribosomal protein S3 antibody was purchased from Santa Cruz Biotechnology (Santa Cruz, CA).

### Cell Lines and Culture

Cell lines were purchased from the Institute of Biochemistry and Cell Biology (Shanghai, China), and maintained according to the suppliers’ instructions. In brief, P388 cells were maintained in Dulbecco’s modified Eagle’s minimum essential medium (DMEM); A459 cells were cultured in F12K medium. Both media were supplemented with 10% fetal bovine serum. The cells were maintained in 5% CO2 at 37°C.

### Cell Proliferation Assay

The cell proliferation was measured by SRB assay. P388 cells were cultured at 8×10^3^ cells/well in 96-well plates and then treated with UP for 72 h. In some experiments the cells were pretreated with NAC, iminazole or MAPK inhibitor for 1 h. The cells were incubated with 16% trichloroacetic acid (TCA) at 4°C for 1 h, after which, plates were washed five times with cold water, the excess water was drained off and the plates left to dry in air. SRB stain (100 µl, 0.4% in 1% acetic acid) was added to each well and left in contact with the cells for 30 min, then the cells were washed with 1% acetic acid for five times. The plates were dried and 150 µl of 10 mM Tris base (pH 10.5) was added to each well to solubilise the dye for 30 min. The absorbance (OD) of each well was read on a Microplate Reader (Tecan, Austria) at 490 nm wavelength. The% of cell viability = (OD of treated cells/OD of control cells)× 100%.

### DNA Content Analysis

P388 cells were plated in 100 mm culture dish at 2×10^5^ cells/dish, 24 h later, cells were treated with UP for 24 h. After that, the cells were harvested, fixed with 70% cold ethanol at 4°C for 12 h, incubated with RNase A (30 µg/ml) for 30 min at room temperature, and then stained with propidium iodide (50 µg/ml) at 4°C for 30 min. Cellular DNA was analyzed by flow cytometry (Vantage, Becton Dickinson, San Jose, CA).

### Western Blot Analysis

After UP (0.05 µM) treatment for 1 h or 24 h, 1×10^6^ cells were washed with PBS and then lysed with RIPA (50 mM Tris, pH 7.4, 150 mM NaCl, 1% NP-40, 0.5% sodium deoxycholate, 0.1% SDS) for 30 min on ice. The cell lysate was loaded for electrophoresis in 10% SDS-PAGE. The separated proteins were electrophoretically blotted onto NC membrane (millipore, USA). The membranes were blocked in 5% non-fat milk diluted in TBS-T for 2 h at RT, then incubated overnight with antibodies for the PARP, Cyt C, Caspase-8, 9, 3, p-AKT, AKT, p-ERK, ERK, p-JNK, JNK, p-P38 and P38. Goat anti-rabbit IgG conjugated to horseradish peroxidase (1∶3000 dilution) were incubated for 1 h at RT. Between each incubation, the membranes were washed 3×5 min in TBS-T. Antibody binding was detected with enhanced chemiluminescence reagent.

### Localization of UP in Cells

P388 cells grown on coverslips were treated with UP (0.05 µM).at 37°C for 1 h. After three washes with PBS, cells were then incubated with DAPI. The coverslips were examined with a laser scanning confocal microscope (LSM510-Meta, Zeiss, German) [10].

### AO Staining

The living cultured cells were stained with acridine orange as described previously [11], [12]. Briefly, P388 cells on a chamber slide were exposed to UP for 1 h. Then the cells were incubated with 5 µg/ml acridine orange for 30 min at 37°C in the dark. The chamber slides were washed with Hanks’ solution and then were examined using confocal laser microscopy.

### Detection of Intracellular pH (pHi)

P388 cells were exposed to 0.05 µM of UP for 1 h. Cultured cells were washed with PBS, centrifuged and loaded with 10 µM 29, 79-bis-(carboxyethyl)-5(69)-Carboxyfluorescein acetoxymethyl ester (BCECF-AM) at 37°C for 30 min in PBS. After centrifugation, cells were re-suspended in PBS and analyzed by flow cytometry [4].

### Detection of Intracellular Reactive Oxygen Species (ROS)

Intracellular oxidative stress was assessed by measuring intracellular oxidation of 2′, 7′-dichlorofluorescin (DCFH). The substrate is DCFH-DA which easily diffuses into the cell and is next deacetylated by cellular esterases to the more hydrophilic, nonfluorescent DCFH. ROS generation in the cell oxidizes DCFH to the fluorescent 2′, 7′-dichlorofluorescein (DCF). P388 cells were seeded, and incubated with UP (0.05 µM ) for 1 h, then harvested cells, washed, and loaded with DCFH (10 µM ) at 37°C for 30 min. Then the cells were harvested, washed, and loaded with DCFH (10 µM ) at 37°C for 30 min. Finally the cells were washed with PBS, and cellular fluorescence was acquired using flow cytometry with excitation at 488 nm and emission at 530 nm [13], [14].

### Native–PAGE and Mass Spectrometric Analysis

After incubation with UP (0.05 µM) for 1 h at 37°C, P388 cells were harvested and lysed with lysis buffer (20 mM Tris, pH7.5, 150 mM NaCl, 1% Triton X-100 ) for 30 min on the ice, centrifuged at 12000 g for 20 min at 4°C. An equal volume of loading buffer(50 mM trisbase, 30% glycerol, 0.01% bromophenol blue) was added to supernatant. The samples were separated on 8% Native–PAGE, and the strip was cut according to the color of UP. The proteins binding with UP were analyzed by LC-ESI-LTQ (Thermo Finnigan LTQ) against NCBI mouse protein database [15].

### Sucrose Density Gradient Fractionation

To prepare cytoplasmic extract for ribosomal fractionation, P388 cells were treated with 0.05 µM UP for 1 h, then washed with ice-cold PBS twice and lysed in ice-cold RIPA for 30 min, then centrifuged at 10,000 ×g for 15 min to clear the resultant supernatant of nuclei, mitochondria and debris. Lysate protein solution was layered over 9 ml linear sucrose gradient solution (10–50%) in a 11.5 ml Sorvall centrifuge tube and centrifuged at 35,000 ×g for 3 h at 4°C in ultracentrifuge (Beckman Optima tm L-100 XP). Only UP without lysate on top of linear sucrose gradient solution was used as control [16].

### Immunofluorescence Staining

P388 cells were inoculated in a six-well plate and cultured overnight. Cells were exposed to UP (0.05 µM) for 1 h. Cells were rinsed twice with phosphate-buffered saline (PBS) and fixed for 10 min with 4% formaldehyde. Next, the cells were washed twice with PBS and then permeabilized for 10 min with 0.1% Triton X-100, the cells were pre-incubated with PBS containing 1% BSA for 20–30 min and stained with ribosomal protein S3 antibody overnight at 4°C, followed by incubation with anti-mouse IgG-FITC for 30 min. The cells were then washed with PBS and examined using confocal laser-scanning microscopes [17].

## Results

### UP Inhibits the Growth of P388 Cells

**Figure 2:**
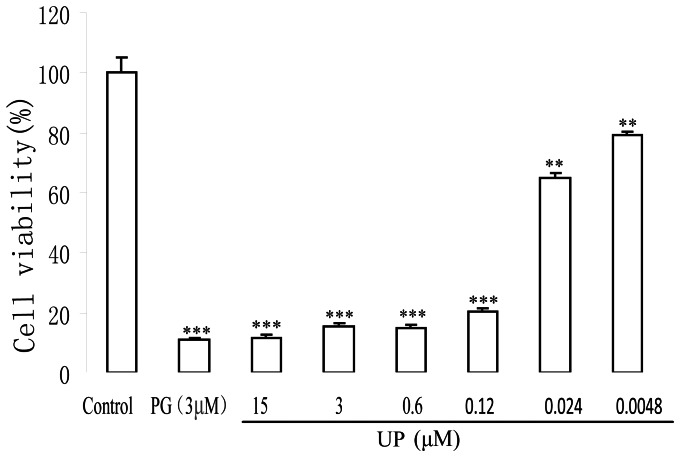
Effect of UP on the growth of P388 cells. Cells were treated with indicated concentration of UP and PG for 72 h. The data shown represent the percentages of cell viability of three independent experiments with three determinations in each. **, *p*<0.01 and ***, p<0.001 vs. Control.

To determine the cytotoxic effect of UP on P388 cells, SRB assay was used to assess cell proliferation after 72 h treatment. In a concentration range of 0.0048 to 15 *μ*Μ, UP treatment resulted in a concentration-dependent inhibition in cell growth of P388 cells. The IC_20_ and IC_50_ on P388 cells were 0.0049 *μ*Μ and 0.042 *μ*Μ respectively (Fig. 2).

### UP Induces G2/M Phase Arrest and Apoptosis in P388 Cells

**Figure 3:**
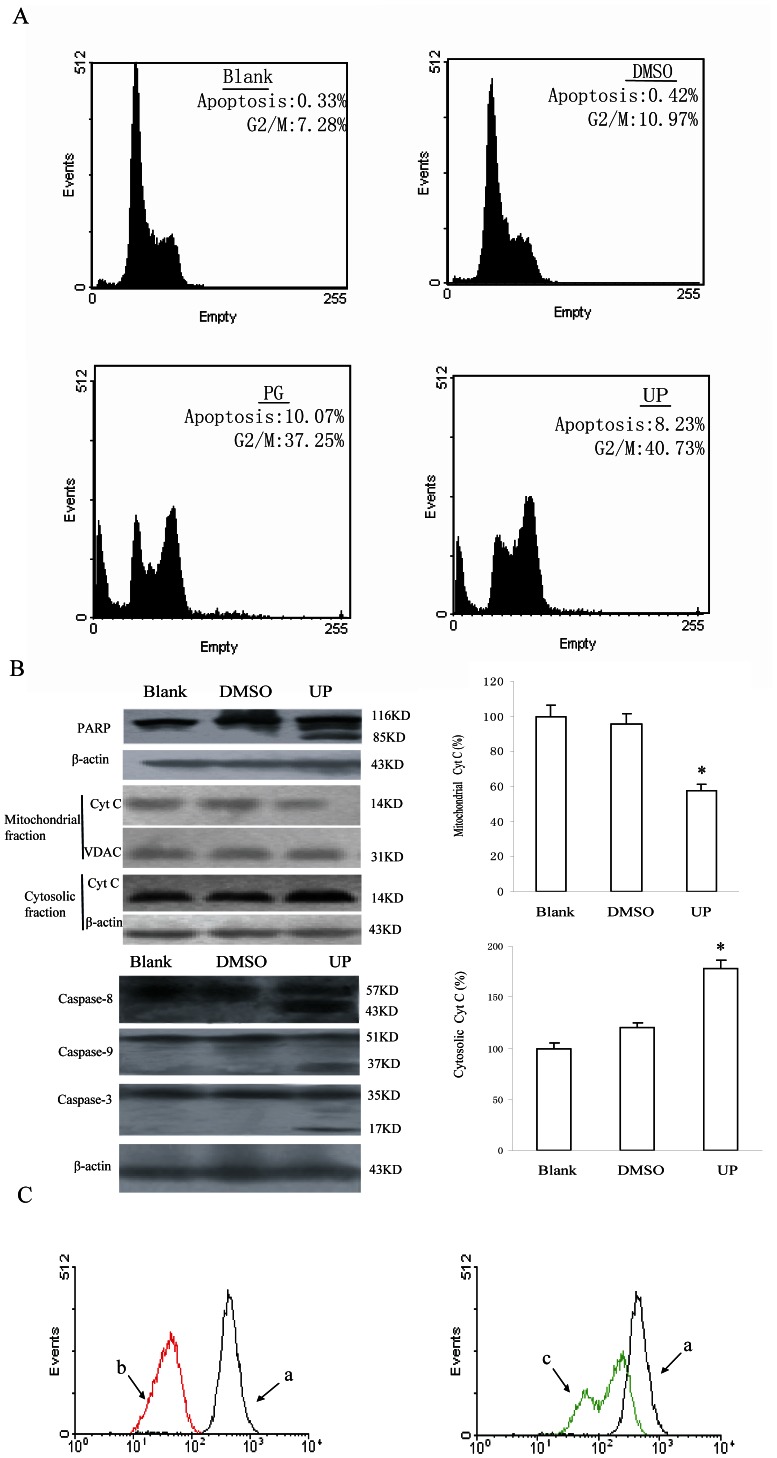
UP induces apoptosis and G2/M phase arrest in P388 cells. A. DNA histograms of P388 cells cultured in medium alone, or in DMSO (1∶1000) or in the presence of 0.05 µM PG and UP for 24 h. B. Expression of apoptosis-related proteins was determined by immunoblotting with specific antibodies following exposure to UP for 24 hr. The expression was quantified using the computerized image analysis system ImageQuant. Each column represents those scanned in duplicate of three wells expressed as percentage of control ± Standard Deviation. *, *P*<0.05, UP vs. Blank. C. Representative flow cytometry profiles by Rhodamine 123 staining, after the cells were incubated with 0.05 µM of UP for 24 h. a, DMSO; b, 0.05 µM PG; c, 0.05 μΜ UP.

The suppression of cancer cell growth has been known to be primarily mediated by apoptosis and cell cycle arrest. We next applied flow cytometry to analyze cell cycle phase distribution and apoptosis upon UP treatment. P388 cells treated with UP (0.05 µM) for 24 h showed accumulation in G2/M (40.73%) with a concomitant decrease in G0/G1 phase cells, suggesting a cell cycle arrest at G2/M phase. UP also induced a higher percentage of sub G0/G1 population, indicative of apoptosis occurrence. About 8.23% cells was apoptotic when treated with UP for 24 h (Fig. 3A) in contrast to <1% apoptotic cells in the untreated control group. Further, we examined UP induced poly (ADP-ribose) polymerase cleavage, a hallmark of apoptosis that indicates activation of caspases. The results showed that hydrolysis of the 116 KD poly (ADP-ribose) polymerase protein to the 85 KD was detected in UP treated cells after 24-h exposure at 0.05 µM. To examine whether caspases were involved in UP induced apoptosis, western blot analysis was carried out to further confirm the participation of caspase-3, caspase-8 and caspase-9. The cleavaged bands of procaspase-3, procaspase-8 and procaspase-9 were detected after treatment with UP for 24 h, indicating the activation of caspase-3, caspase-8 and caspase-9.

We next examined the mitochondrial release of Cyt C, the upstream pathway of caspase 9. After 24-h treatment with UP, the release of Cyt C in the cytosol was considerably increased, whereas Cyt C in the mitochondria decreased obviously (Fig. 3B). It has been know that release of intermembrane proteins like Cyt C occurs after the integrity of mitochondrial membrane was impaired, which leads to a disruption of the inner transmembrane potential (ΔΨm). The impact of UP on mitochondrial transmembrane potential (ΔΨm) was further assessed using rhodamine 123, a specific fluorescent probe. Fluorescent intensity was significantly decreased in UP treated cells, compared with the control group (Fig. 3C). These results together suggest that the intrinsic mitochondrial apoptotic pathway, involving caspase-9 and -3, is implicated in UP-induced apoptosis in P388 cells.

### UP Predominantly Localizes in Cytoplasm

**Figure 4:**
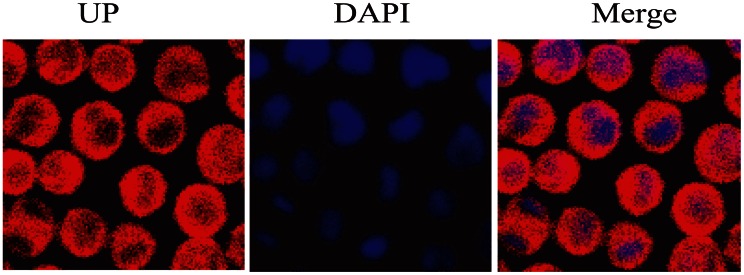
Confocal fluorescence images of P388 cells incubated with 0.05 µM UP for 1 h. The blue represents the nucleus staining and the red represents UP.

UP emits red fluorescence at 488 nm excitation, which can be visualized in living cells. P388 cells were stained with the DNA binding dye DAPI after 1 h exposure to 0.05 µM UP to examine the sub-cellular localization of UP. The cells were imaged under confocal laser microscopy. As shown in Fig. 4, UP was predominantly localized in the cytoplasm, but not membrane or in the nucleus.

### UP Activates MAPK but not AKT Signaling

**Figure 5:**
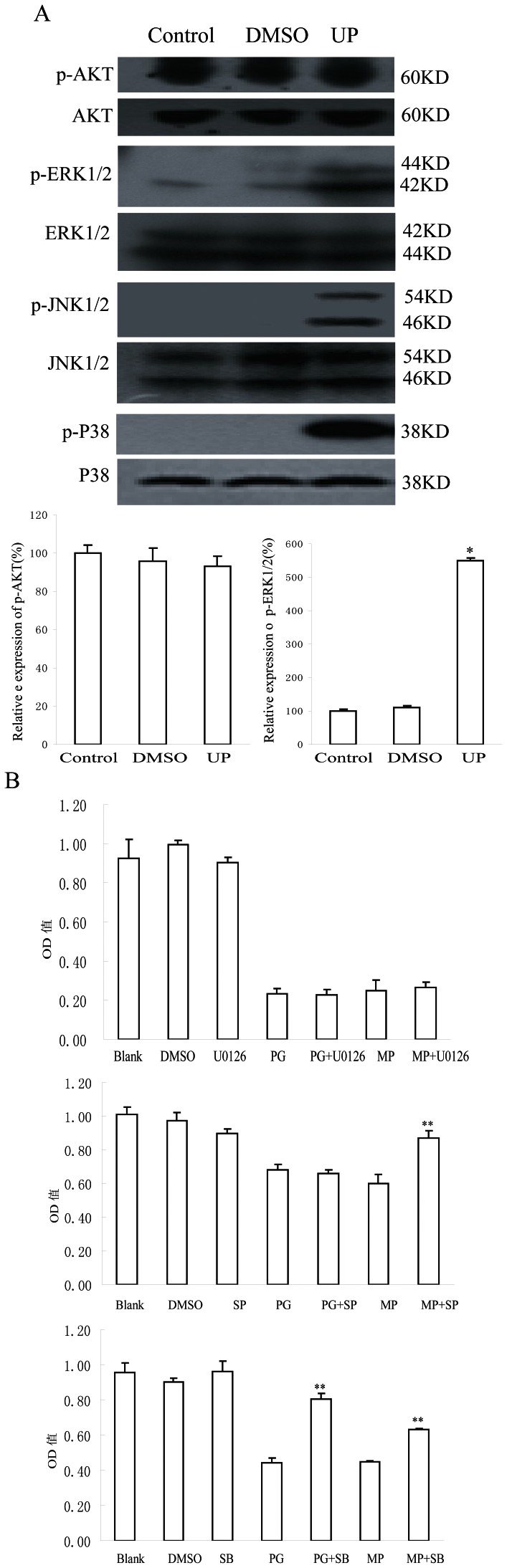
Activated protein kinases related to UP induced apoptosis. **A**. Effects of exposure of P388 cells to 0.05 µM UP for 1 h on the phosphorylation status and expression level of protein kinases. The expression was quantified using the computerized image analysis system ImageQuant. Each column represents those scanned in duplicate of three wells expressed as percentage of control ± Standard Deviation. ***, *P*<0.001, UP vs. Control. **B**. Effects of MAPK inhibitors on UP-induced P388 growth inhibition,the cells were pretreated with U0126 (10 µM), SP60012 (20 µM) or SB203580 (20 µM) for 1 h, then co-treated with UP for 72 h. U0126, ERK inhibitor; SP60012(SP), JNK inhibitor; SB203580(SB), P38 inhibitor. **, P<0.01, PG vs.(PG+inhibitor),UP vs.(UP+ inhibitor).

To determine the potential involvement of protein kinase pathways in G2/M arrest and apoptosis induced by UP, we probed the phosphorylation status of four major protein kinases which relate to cell proliferation in P388 cells after exposed to UP for 1 h. As shown in Fig. 5A, UP obviously increased the levels of phospho-ERK1/2, phospho-p38MAPK, and phospho-JNK1/2, whereas AKT phosphorylation status was barely affected. Total protein content of the respective protein kinases was not changed.

To further address whether ERK1/2, JNK1/2 or P38 was involved in UP-inhibited cell growth, cells were incubated with or without the inhibitor of ERK1/2, JNK1/2 and P38 respectively and then cotreated with 0.05 µM UP for 72 h. Cells proliferation was estimated by the SRB method. As shown in Fig. 5B, none of these inhibitors affected the cells proliferation per se. SP and SB but not U0126 obviously reversed the inhibitory effect of UP on proliferation of P388 cells, indicating that JNK1/2 and P38 activation was involved in UP-induced apoptosis. Only P38 activation was found to be related to PG-induced apoptosis, which was in agreement with literature reports [18].

### NAC Fails to Rescue UP Caused Inhibition of Cell Proliferation

**Figure 6:**
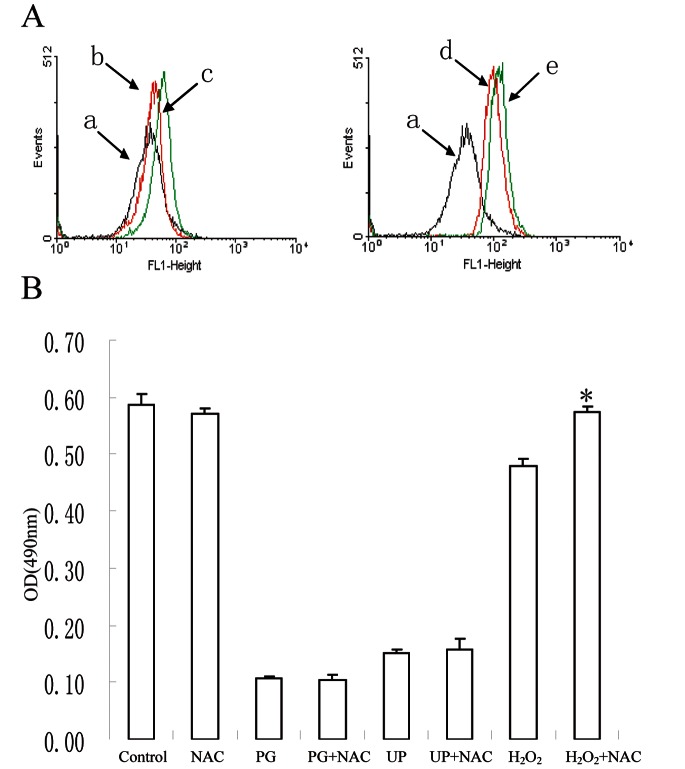
UP causes ROS generation, which is most likely not involved in UP-induced apoptosis. A. P388 cells were treated with 0.05 µM UP for 1 h, and stained with DCFH (10 µM). Fluorescence was measured by flow cytometry. a, DMSO+DCFH; b, 0.05 µM PG; c, 0.05 µM PG +DCFH; d, 0.05 µM UP; e, 0.05 µM UP+DCFH. B. P388 cells were pretreatment with or without NAC (0.5 mM) for 1 h, then cells were co- incubated with PG (0.05 µM ), UP (0.05 µM) and H_2_O_2_ (0.4 mM) for 72 h. *, P<0.05, H_2_O_2_ vs (H_2_O_2_+NAC).

It is reported that PGs could induce the ROS production in cells [19]. To examine the possible the involvement of ROS in UP-induced apoptosis and G2/M phase arrest in P388 cells, we firstly measured ROS generation in the cells. The results showed that ROS generation was increased as early as 1 h after UP treatment (Fig. 6A). To reveal whether the ROS generation accounts for UP-induced apoptosis, a ROS scavenger NAC was used prior to UP treatment. As shown in Fig. 6A, NAC failed to rescue the growth inhibition induced by UP and PG, where in contrast H_2_O_2_ suppressed cell proliferation was clearly reversed by NAC treatment. These findings suggest that ROS generation is most likely not involved in UP-induced apoptosis.

### Acidification does not Participate in UP Caused Growth Inhibition

**Figure 7:**
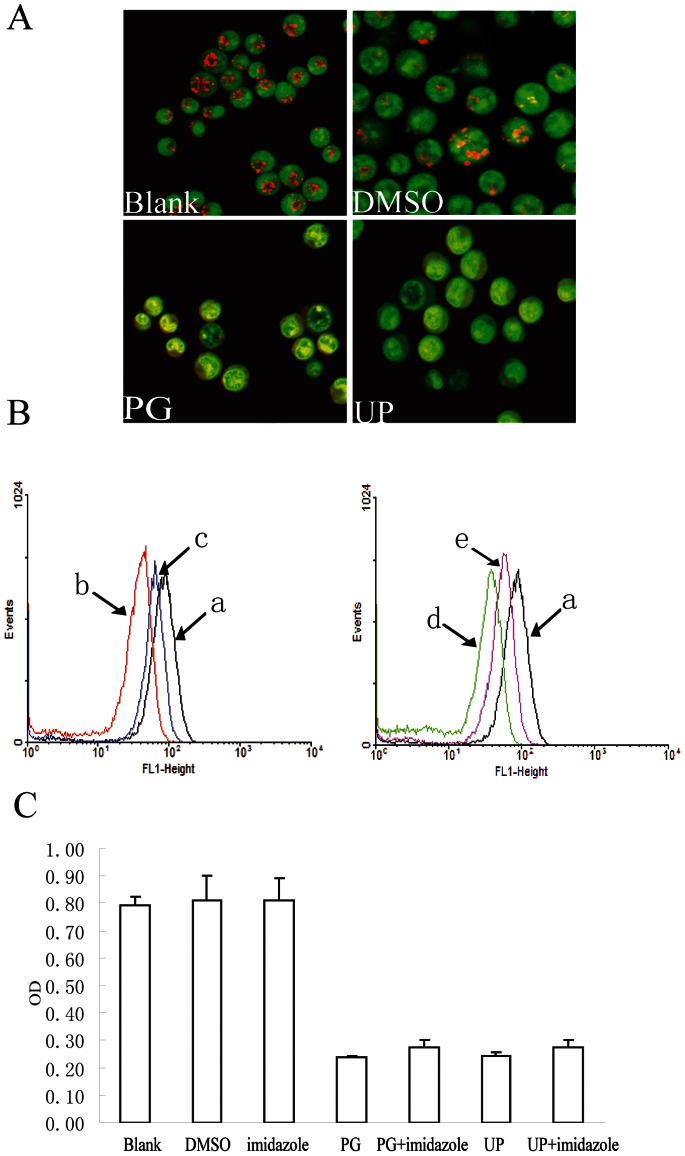
Acidification does not participate in UP caused growth inhibition. A. Acridine orange staining of P388 cells, PG and MP were 0.05 µM, AO was 5 µg/ml. Orange granules were acidified compartments. B. Represent data of flow-cytometric pHi analysis using 10 µM BCECF-AM staining after the cells were incubated with 0.05 µM of PG or UP for 24 h with or without imidazole (0.5 mM) pretreatment for 1 h. a, Control; b, PG; c, PG+imidazole; d, UP; e, UP+ imidazole C. Effect of acidification inhibitor on UP-induced P388 growth inhibition, the cells were pretreated with imidazole (0.5 mM ) for 1 h, then co-treated with PG (0.05 µM) and UP (0.05 µM) for 72 h.

Acridine orange is an “acid- tropic” weak base, which is taken up by living cells and accumulates in acidified compartments such as lysosomes [17], [20]. Fluorescence of acridine orange is green at low concentrations, whereas at high concentrations the fluorescence changes to orange [17]. We observed obvious disappearance in orange granules in cells after short treatment (1 h) with 0.05 µM of PG and UP (Fig. 7A). The changes of pHi were also analyzed by flow cytometry with BCECF-AM, a membrane-permeable fluorescent indicator for the measurement of cytoplasmic pH. We found that the pHi decreased considerably in PG or UP-treated cells (Fig. 7B) compared with untreated cells.

In contrast, cell-permeable bases imidazole treatment markedly prevented the decrease in pHi. We then tested whether UP-caused growth inhibition could be rescued by imidazole. Concurrent to UP exposure, we weren’t able to identify any significant differences compared with cells treated, about only 3% recovery (Fig. 7C), suggesting that acidification of cytoplasm should not be the main reason of apoptosis induced by UP. PG was noticed to have similar results to UP.

### UP Binds to the Ribosome in P388 Cells

**Figure 8:**
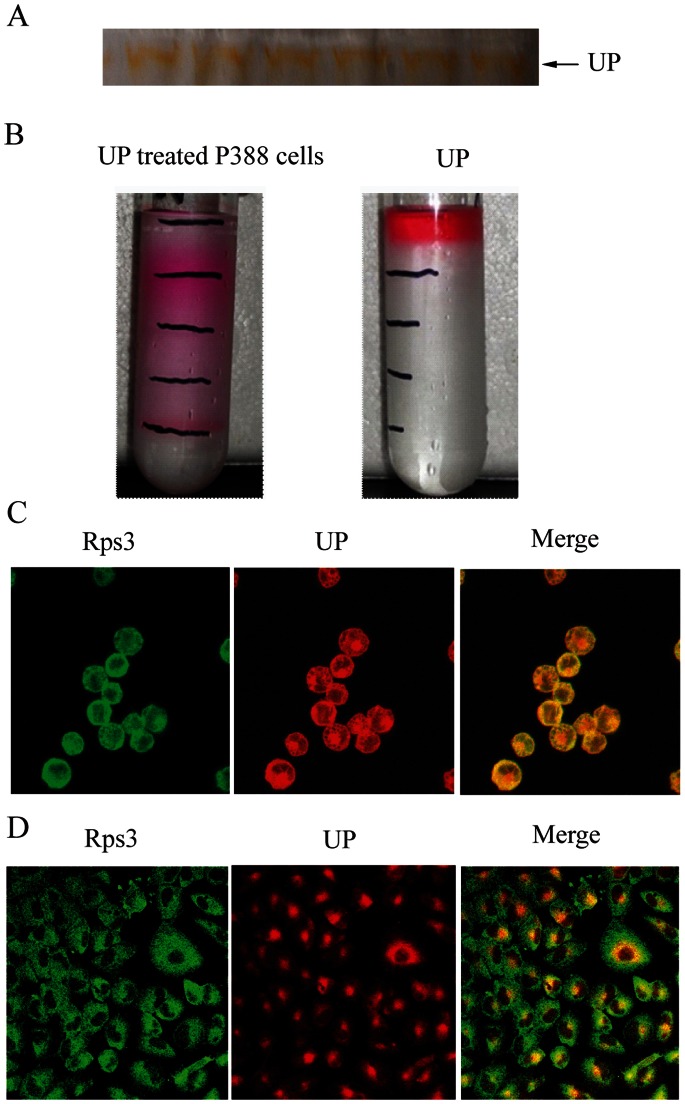
UP binds to the ribosome. **A**. The strip of UP in Native–PAGE. **B**. Sucrose density gradient fractionation of UP-treated P388 cells and UP alone. **C and D.** Subcellular localization of Rps3 and UP in P388 cells (C) and A549 cells (D) detected by confocal. UP fluorescence is shown in red, Rps3 fluorescence in green and the colocalization (merge) in yellow.

We proceeded to identify the molecular targets of UP in p388 cells by identifying UP binding proteins using mass spectrometry analysis. UP migration can be easily observed with an unaided eye as a band in orange color in native PAGE (Fig. 8A). The gel band was cut and subjected to mass spectrometry analysis. A total of 951 proteins were detected, amongst which 171 proteins, to our surprise, were ribosomal proteins with higher CoverPercent (Table 1), suggesting that UP may primarily bind to ribosome.

**Table 1:** Ribosome proteins binding to UP identified by MS.

Number	Protein	CoverPercent	
1	Rps3 40S ribosomal protein S3		63.79%
2	Rps8 40S ribosomal protein S8		50.96%
3	Rpl23 Ribosomal protein L23		46.67%
4	Eef1a1 Elongation factor 1-alpha 1		41.56%
5	Rps17 40S ribosomal protein S17		41.48%
6	Rps13 40S protein		41.27%
7	Eef2 Elongation factor 2		38.81%
8	Rps18 40S ribosomal protein S18		32.24%
9	Gm9493 similar to ribosomal protein S7		31.29%
10	Rps3a 40S ribosomal protein S3a		31.06%
11	Eef1g Elongation factor 1-gamma		28.60%
12	Rps5 ribosomal protein S5		28.43%
13	Rpl7 60S ribosomal protein L7		26.67%
14	Rpl17 60S ribosomal protein L17		26.63%
15	Rps7 40S ribosomal protein S7		26.29%
16	Rps11 40S ribosomal protein S11		25.95%
17	Eef1d Isoform 1 of Elongation factor 1-delta		23.13%
18	Rps20 40S ribosomal protein S20		22.69%
19	Rpl9;Gm10321 60S ribosomal protein L9		22.4%
20	Rpl18a 60S ribosomal protein L18a		21.59%
21	Rpl23 60S ribosomal protein L23		20.00%

For further validation, P388 cells were incubated with UP at 0.05 µM for 1 h and ribosome was isolated by sucrose density gradient fractionation. Comparing with control group where UP was enriched at top of sucrose density solution, obvious layered color-bands were found in UP treated cells (Fig. 8B), supporting a notion that UP binds to ribosome. We also detected the colocalization between UP and ribosome using confocal microscope in both p388 cells (Fig. 8C) and A549 cells (Fig. 8D). Immunofluorescence staining showed that UP colocalized to ribosome indentified by endogenous Rps3 in the two cell lines.

## Discussion

Up to date, the anticancer mechanism of PGs still have not been clarified in details. For example, Francisco R reported PG impacted mitochondria, exerted an uncoupling effect on the electronic chain, the cell nucleus was preserved from prodigiosin acces**s** [17]. However, the findings of Montaner B indicated that apoptosis induced by PGs was copper-mediated cleavage of DNA [21]. Jing Zhang et al. also reported that PGs increased intercellular ROS, which led to cytotoxic effects [19]. The molecular targets of PGs are inconsistently reported including JAK3, mTOR and NAG-1 [7], [8], [22]. In our study, we think that UP induced apoptosis in P388 cells is associated with the UP-ribosome binding.

We found that UP inhibited the cell proliferation, arrested the cell cycle at G2/M phase, led to activation of caspase 3, 8, 9, alterations of mitochondria membrane potential, release of Cyt C and cleavage of PARP. These results indicate that UP has cytotoxicity to P388 and induces P388 cells apoptosis involving in mitochondria apoptotic pathway at least. In order to elucidate the mechanism underlying apoptosis induced by UP, we first assessed the cell distribution of UP with its auto-fluorescence, the result showed that UP distributed in cytoplasm but not membrane or nucleus, which suggests us that UP may interact with molecules in cytoplasm to induce apoptosis.

The PI3K/Akt pathway regulates fundamental cellular functions such as cell growth, survival, and movement. Extravagant activation of the PI3K/Akt pathway has been reported to be associated with the development of cancer. Akt can also directly regulate the apoptotic machinery by phosphorylating and inactivating pro-apoptotic proteins such as BAD, in addition, Akt activity is required for G2/M phase transition and AKT inhibition often induces G2-M arrest [23] **.** However, in our experiments, UP wasn’t found to influence AKT activation of P388.

MAP kinases are composed of serine/threonine protein kinases, including the extracellular signal-regulated kinases (ERK), the p38 MAPK, the c-Jun-NH2-terminal kinases (JNK), and MAP kinases signaling pathways regulate essentially all aspects of malignant cell behavior, proliferation, survival, migration and invasion [24]. Our results showed that UP activates P38, ERK and JNK remarkably. The selective inhibitors of P38 and JNK rather than ERK inhibitor, prevented the P388 cancer cells from cytotoxicity induced by UP. These results indicate that apoptosis induced by UP is related to activation of P38 and JNK, which is different from PG by which only phosphorylation of p38 involves in its activity.

ROS is generated intracellularly as byproducts of normal aerobic metabolism or as second messengers in various signal transduction pathways or in response to environmental stress [25], [26]. As specific second messengers in signaling cascades involved in cell proliferation and differentiation, ROS has been implicated in the regulation of diverse cellular functions including intracellular signaling transcriptional activation, proliferation and apoptosis [27]. In recent years, many research focus on the relationship between ROS and mitogen activated protein kinase-MA*PK* signaling pathways. Ling Liu et al reported that NG-induced apoptosis of HepG2 cells was characteristic of intracellular ROS generation. Simultaneously NG treatment could lead to the activation of the phosphorylation of JNK and p38 but not ERK1/2. Our data demonstrated that UP induced intracellular ROS production in P388 cells. However, a ROS scavenger NAC could not reverse inhibition of proliferation caused by UP, although it obviously antagonized the ROS production by H_2_O_2_. These results indicate that generation of ROS is not implicated in apoptosis induced by UP.

The pHi within acidic organelles are responsible for a wide variety of important cellular functions, such as endocytosis, exocytosis and intracellular trafficking, as well as cell differentiation, cell growth and cell death. The pHi in transformed or cancerous cells generally remains neutral or even slightly more alkaline than normal cells [28], regulated by a variety of pHi homeostatic mechanisms, including Na^+^/H^+^, Na^+^-dependent and-independent Cl^−/^HCO_3_^−^ exchangers, vacuolar type H^+^-ATPase (V-ATPase) and others. Daigo Ya mamoto reported that the intracellular acidification of KPL-1 by cPrG.HCl treatment induced apoptosis and cycle arrest, which was strongly suppressed by imidazole, a cell-permeable base. It has been demonstrated that Bafilomycin A1, a potent selective inhibitor of vacuolar H^+^-ATPase [29] also induces a decrease in intracellular pH and inhibits the growth of different cancer cells lines [30]. We also found that UP could decrease intracellular pHi detected by confocal and flow cytometry respectively. However, imidazole, an inhibitor of acidification failed to rescue the growth inhibition of UP. These results rule out the possibility of acidification in apoptosis induced by UP.

Taking advantage of its autofluorescence feature, we noticed that UP is mainly distributed in cytoplasm. We further isolated the proteins binding to UP in native-PAGE gel and submitted to mass spectrometry analysis. 171 proteins from 951 detectable proteins were ribosome-related, suggesting that UP can likely bind to ribosome. We further validated the hypothesis by sucrose density gradient fractionation method, a conventional approach to isolate and study ribosome and by immunofluorescence staining to observe colocalization of UP and ribosome in p388 cells and A549 cells. Ribosomal proteins play multiple roles in coordinating protein biosynthesis to maintain cell homeostasis and survival. Recent evidence suggests that a number of ribosomal proteins have secondary functions independent of their involvement in protein biosynthesis. These proteins function as cell proliferation regulators and in some instances as inducers of cell death [31], [32]. Johnson CR found that interference with 28S-rRNA by BCS initiated apoptosis in REH cells through recruitment of SAPK-JNK signaling [33]. Bae HK reported that SG specifically interacted with 40S and 60S ribosomal subunits as early as 5 min in RAW 264.7 cells and induced phosphorylation of p38 and JNK [16]. Considering that UP could induce P388 cells apoptosis involving the activation of P38 and JNK in our previous study, we speculate that apoptosis of induced by UP may be related to UP-ribosome binding, which in turn affects the function of some ribosome proteins and leads to apoptosis. It was also worthwhile to mention that only two mitochondria proteins were detected by mass spectrometry with coverprecent of protein <10%, which largely excludes the possibility of direct targeting to mitochondrial proteins by UP.

In summary, our results presented in this study exclude the involvement of ROS and acidification in UP induced apoptosis, in spite of tremendous literatures. Our data instead suggests that UP binds to ribosome, which may, at least partially, trigger the P38, JNK signaling and mitochondrial pathway apoptosis, eventually leading to inhibition of cell proliferation.The targeted ribosomal molecules needs to be further studied. Nevertheless, ribosome could possibly open a new avenue for exploring anticancer mechanism of PGs in the future.
