# Correction: Undecylprodigiosin Induced Apoptosis in P388 Cancer Cells Is Associated with Its Binding to Ribosome

**DOI:** 10.1371/journal.pone.0236282

**Published:** 2020-07-14

**Authors:** Ping Liu, Yuan-yuan Wang, Xin Qi, QianQun Gu, Meiyu Geng, Jing Li

After this article [1] was published, concerns were raised about Fig 3B. Specifically, the Blank and DMSO lanes of the Cyt C and VDAC panels for the Mitochondrial fraction appear similar, when rotated 180 degrees.

**Fig 3:**
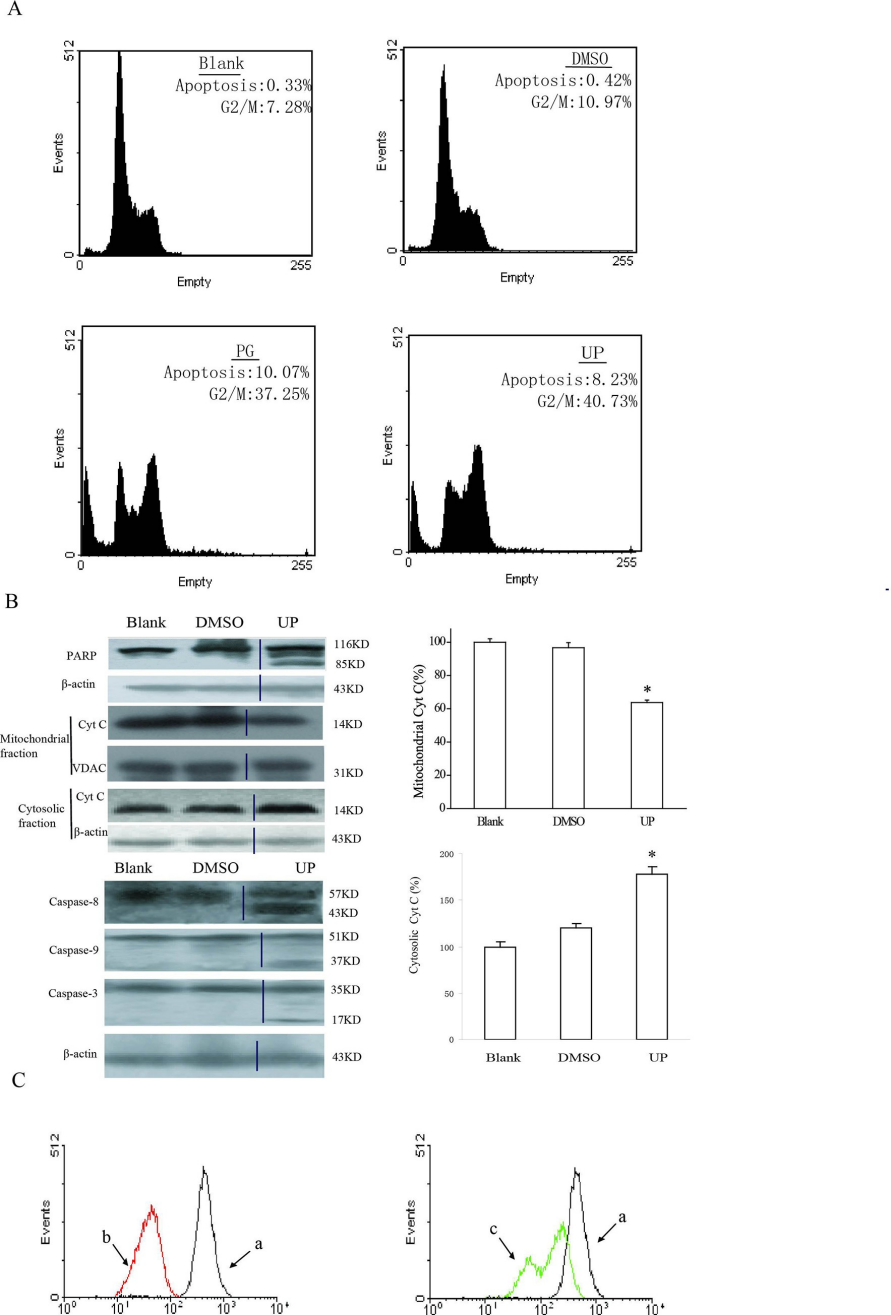
UP induces apoptosis and G2/M phase arrest in P388 cells. A. DNA histograms of P388 cells cultured in medium alone, or in DMSO (1:1000) or in the presence of 0.05 μM PG and UP for 24 h. B. Expression of apoptosis-related proteins was determined by immunoblotting with specific antibodies following exposure to UP for 24 hr. The uncorrelated lanes were cut away and then combined lanes of UP to the lanes of Blank and DMSO. The vertical black lines indicated where the lanes were combined. The expression was quantified using the computerized image analysis system Image Quant. Each column represents quantified intensity in duplicate of three times expressed as percentage of control ± Standard Deviation. *, P < 0.05, UP vs. Blank. C. Representative flow cytometry profiles by Rhodamine 123 staining, after the cells were incubated with 0.05 μM of UP for 24 h. a, DMSO; b, 0.05 μM PG; c, 0.05 μM UP.

The authors commented that these experiments were repeated about three times at the time of the original experiments, and this duplication in the published figure may have arisen due to errors in preparing the figure. The authors have provided replacement data from a different replicate of the Mitochondrial fraction CytC and VDAC blot experiments, along with an updated version of the graph in Fig 3B showing quantification of the replacement blots. A member of *PLOS ONE*’s Editorial Board confirmed that the replacement blot images support the results and conclusions as reported in the article.

In addition, the authors clarified that in preparing Figs 3 and 5 they spliced a lane out of each blot image between the DMSO and UP lanes. They provided updated versions of these figures and the accompanying legends which indicate where images were spliced and which incorporate the replacement Mitochondrial Cyt C and VDAC data discussed above.

**Fig 5:**
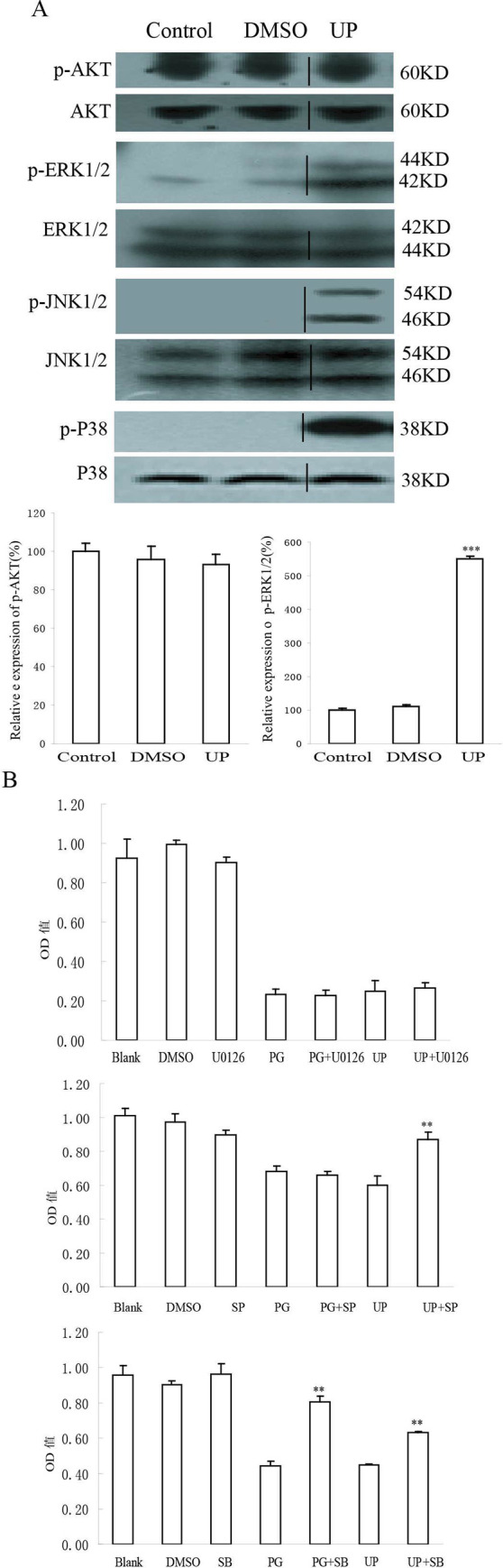
Activated protein kinases related to UP induced apoptosis. A. Effects of exposure of P388 cells to 0.05 μM UP for 1 h on the phosphorylation status and expression level of protein kinases. The uncorrelated lanes were cut away and then combined lanes of UP to the lanes of Control and DMSO. The vertical black lines indicated where the lanes were combined. The expression was quantified using the computerized image analysis system Image Quant. Each column represents quantified intensity in duplicate of three times expressed as percentage of control ± Standard Deviation. ***, P < 0.001, UP vs. Control. B. Effects of MAPK inhibitors on UP-induced P388 growth inhibition, the cells were pretreated with U0126 (10 μM), SP60012 (20 μM) or SB203580 (20 μM) for 1 h, then cotreated with UP for 72 h. U0126, ERK inhibitor; SP60012 (SP), JNK inhibitor; SB203580(SB), P38 inhibitor. **, P < 0.01, PG vs. (PG + inhibitor), UP vs. (UP + inhibitor).

The raw blot images for the Fig 3B replacement panels (Mitochondrial Cyt C and VDAC blots) and the updated quantitative data are in S1 and S2 Files. The authors noted that Fig 8A data are also available, but the original data underlying other results reported in this article are no longer available.

## Supporting information

S1 File Raw images underlying updated Mitochondrial Cyt C and VDAC panels in Fig 3B. (TIF)

S2 File Updated quantitative analysis of Mitochondrial Cyt C results. (DOCX)
