# Supplementary figures and images for: Correction: Undecylprodigiosin Induced Apoptosis in P388 Cancer Cells Is Associated with Its Binding to Ribosome
Source: PLoS One. 2020 Jul 14;15(7):e0236282. doi: 10.1371/journal.pone.0236282 (PMC7360054; doi:10.1371/journal.pone.0236282)

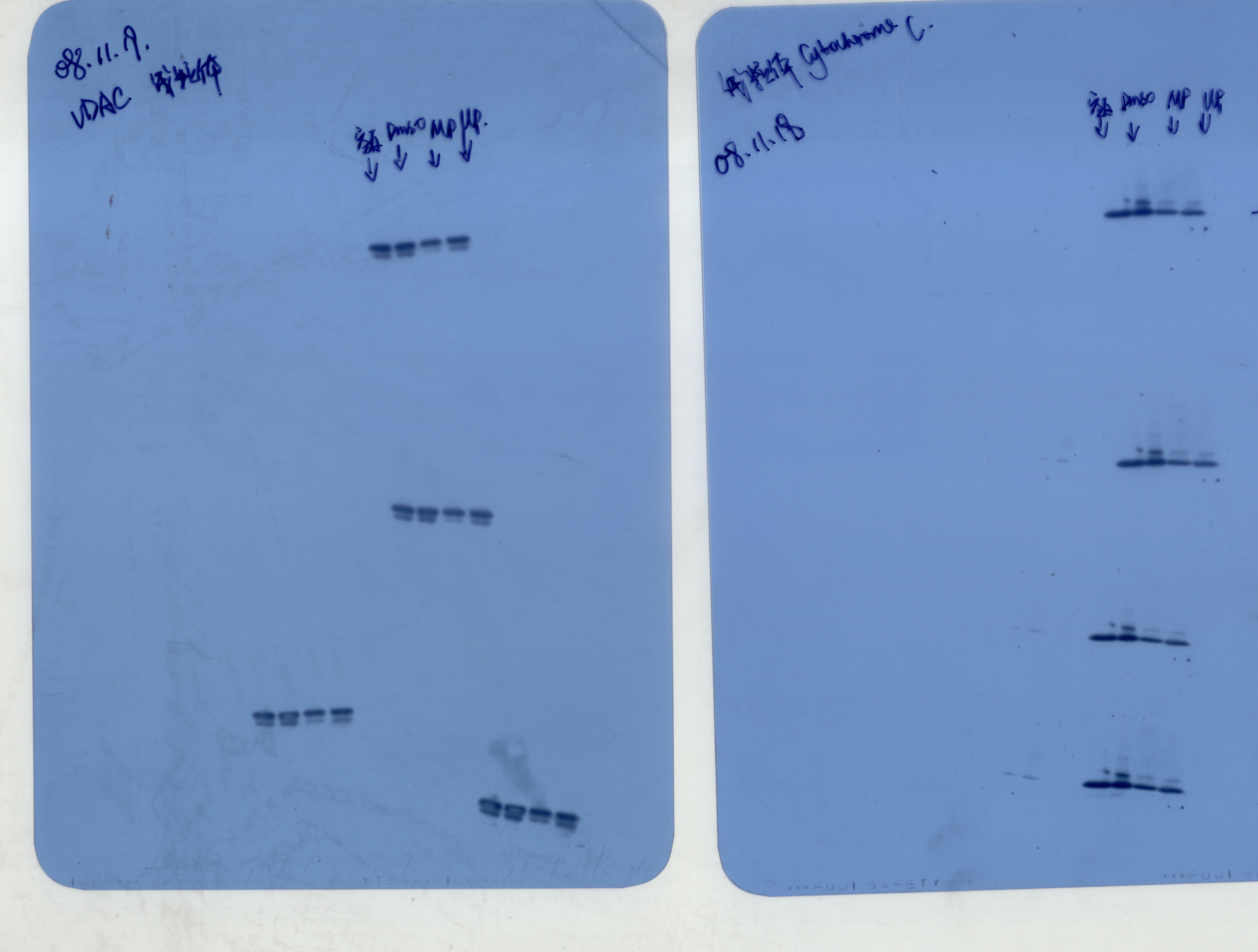

Supplement: S1 File — (TIF) [file pone.0236282.s001.tif]
