# Supplementary material for: Correction: Undecylprodigiosin Induced Apoptosis in P388 Cancer Cells Is Associated with Its Binding to Ribosome
Source: PLoS One. 2020 Jul 14;15(7):e0236282. doi: 10.1371/journal.pone.0236282 (PMC7360054; doi:10.1371/journal.pone.0236282)
Supplement: S2 File — (DOCX) [file pone.0236282.s002.docx]

The bar graph of mitochondrial Cyt C


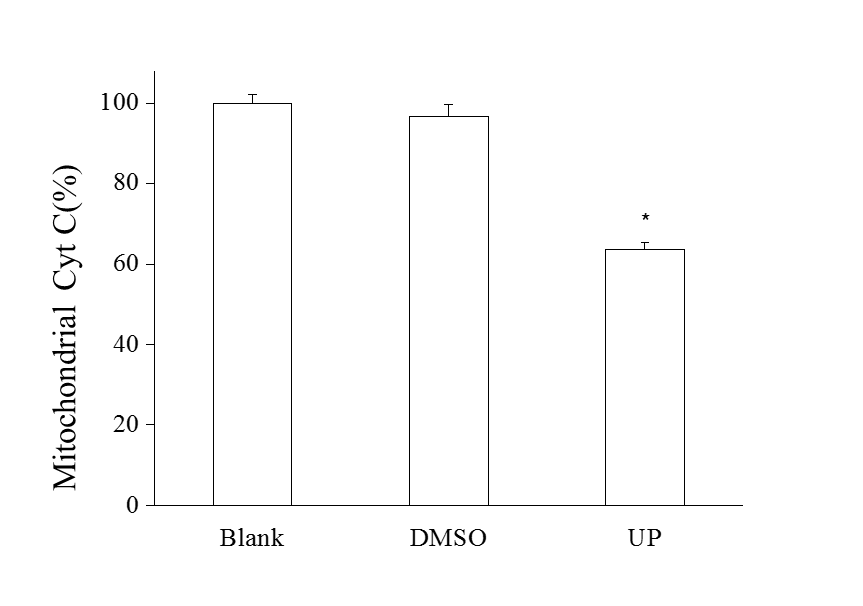


The raw quantitative data of mitochondrial Cyt C and VDAC

|  | **Serial Number** | **Intensity** | | |
| --- | --- | --- | --- | --- |
|  |  | **Blank** | **DMSO** | **UP** |
| **Cyt C** | **1** | **121253** | **118694** | **74655** |
|  | **2** | **115921** | **111034** | **70556** |
|  | **3** | **117304** | **113237** | **73581** |
| **VDAC** | **1** | **115199** | **112637** | **108059** |
|  | **2** | **114557** | **112477** | **109349** |
|  | **3** | **114538** | **116212** | **112395** |
